# Lower-Rim Substituted Calixarenes and Their Applications

**DOI:** 10.1155/2007/65815

**Published:** 2007-03-28

**Authors:** Princy Jose, Shobana Menon

**Affiliations:** Department of Chemistry, School of Sciences, Gujarat University, Navrangpura, Ahmedabad 380009, Gujarat, India

## Abstract

This review discusses in detail “calixarenes” since their discovery as by-products of the phenol formaldehyde bakelites till the present scenario wherein calixarene has assumed a new dimension in the field of supramolecular chemistry. Extensive literature exists for calixarenes; but herein we have tried to concentrate on the different lower-rim modified calixarenes with their potential applications. An attempt has also been made to critically evaluate the synthesis procedures for different lower-rim substituted calixarenes.

## 1. INTRODUCTION

Molecules react chemically in specific and selective ways to form the basis of the living world. Taking cue from this, chemists have shifted their focus from molecular chemistry (chemistry of covalent bonds) to supramolecular chemistry (chemistry of noncovalent interactions) with the supramolecular architecture being constructed either through self-assembly of subunits or by selective host-guest interactions. In self-assembly, particles assemble into atoms, atoms assemble into molecules, and these molecules react with one another to reassemble into new molecules. The molecules assemble with themselves and with other molecules via intermolecular noncovalent bonds to form supramolecular arrays or assemblies [1]. The structural integrity of the final architecture is preserved by noncovalent interactions. The most effective example of self-assembly occurring in living systems is virus, the mechanism of which has been studied in detail. Inspired by the nature's ways of constructing and functioning, the synthetic chemists had the most logical choice of creating supramolecular assemblies via self-assembly. Before the accidental discovery of crown ethers [2] by Pedersen, the word supramolecule was not commonly used; but along with cyclodextrins [3] the crown ethers were also included as another example of host-guest interactions. Later came the discovery of calixarenes [4], which became the third generation of supramolecules. All coexist under the title of supramolecules as well as host-guest chemistry.

Since there is no clear demarcation between host-guest and supramolecule, it is indeed not always possible to differentiate the two, however, molecular recognition is a cornerstone of both these facts. Since the attention is on molecules having molecular cavities which will act as building blocks for the compounds to mimic natural molecular recognition processes, calix[*n*]arenes serve as a good example of study in supramolecular chemistry. As already mentioned above, calixarenes are a widely recognized and researched topic in supramolecular chemistry. There were and there are still a good number of research groups whose work has already generated hundreds of original journal articles, extensive literature reviews [5–9] and monographs [10].

### 1.1. Calixarenes: their origin and synthesis

**Figure 1:**
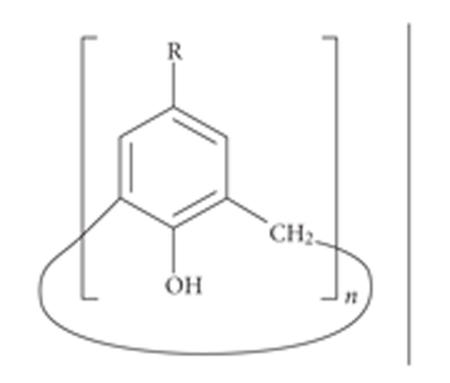
[1_4_]metacyclophane or calix[4]arene.

In 1872, Adolf von Baeyer heated aqueous formaldehyde with phenol to give a hard resinous product. Three decades later, in 1905–1909, Leo Baekland devised a process and he marketed the strong resin obtained from phenol-formaldehyde, under the name Bakelite. Alois Zinke, a new entrant in this field explored the different possibilities of reacting various *p*-alkyl phenols with aqueous formaldehyde and sodium hydroxide, and assigned the products of the treatment as cyclic tetrameric structures calling them “mehrkernmethylenephenolvorbindungen.” These compounds were classed as [1*_n_*]metacyclophanes (where *n* is the number of benzene rings) (Figure 1).

**Figure 2:**
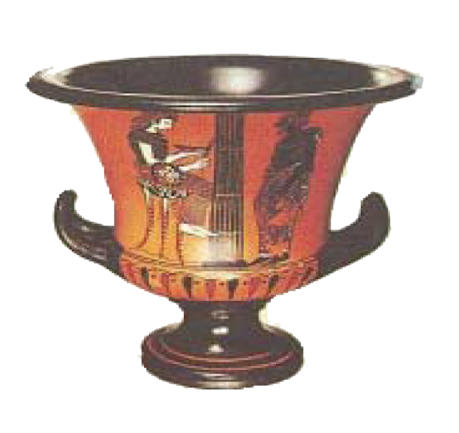
Calix crater.

Although worked upon by many, the credit of naming this class goes to C. D. Gutsche who perceives a similarity between the shapes of these cyclic tetramers and a type of Greek vase known as calix crater (Figure 2); they suggested the compound to be called “Calixarenes.”

**Figure 3:**
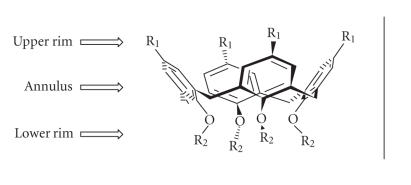
Division of calix[4]arene (applicable to all the calixarenes).

Calixarenes are a class of cyclooligomers formed after phenol-formaldehyde condensation, with defined upper and lower rims and a central annulus (Figure 3).

**Figure 4:**
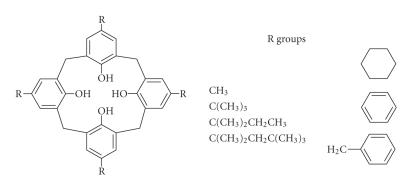
Parent calix[4]arene with different alkyl substituents on the upper rim.

Thus originated a category of compounds derived from *p*-tert-butyl phenol and it was designated as *p*-tert-butyl calix[*n*]arene. After Zinke reported that *p*-methyl, *p*-tert-butyl, *p*-amyl, *p*-octyl, *p*-cyclo-hexyl, *p*-benzyl (Figure 4), and *p*-phenyl phenol condense with formaldehyde to yield high melting materials, all of which the group assumed to be cyclic tetramers, it was later extensively characterized by Kammerer and the interpretation was subsequently invalidated.

**Figure 5:**
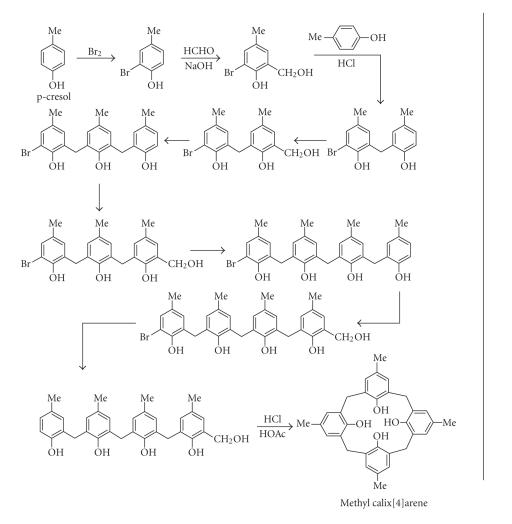
Ten-step synthesis of methylcalix[4]arene by Hayes and Hunter.

The same group modified the 10-step procedure of synthesizing calixarene from *p*-cresol of Hayes and Hunter (Figure 5).

**Figure 6:**
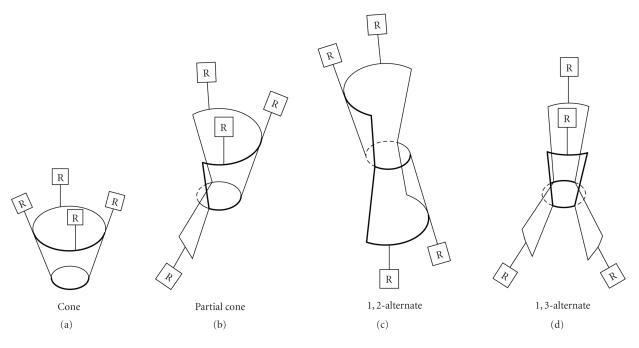
Conformers of calix[4]arenas.

Due to the presence of their preformed cavities, the calixarenes are able to act as host molecules. Due to such structural elaboration, the calixarenes lend themselves well to many applications. This “crater” or “basket” plays a very important role in shaping the entire architecture of calixarene for its function in host-guest chemistry, since this theory is highly interdependent on two terms: “shape” and “functionality.” One of the most interesting and fascinating aspects of calixarenes lies in the fact that they can assume different forms due to the flexibility in the rotation of Ar−CH_2_−Ar bonds, and hence came into existence the “cone,” “partial cone,” “1,2-alternate,” and “1,3-alternate” conformations (Figure 6), which were earlier suggested by Cornforth and later designated by Gutsche [10]. Calix[6]arenes can exist in eight different “up-down” conformations like calix[4]arenes[5], wherein cone conformation is the most stable conformation amongst all the forms of calix[*n*]arenes.

A series of books and reviews have been published which discuss in length the calixarenes and its substituted derivatives being used for the recognition of cation, anion neutral molecules, and organic moieties [1–10]. The literature survey reveals that plenty of work has been done in synthesizing upper-rim modified calixarenes. Comparatively less work has been done at the lower rim of calixarenes. The focus of modifying the calixarenes was much concentrated on the upper rim, due to the easy removal of *t*-butyl group that facilitated different substituted calixarenes [4, 10] with many applications.

## 2. STRUCTURAL MODIFICATIONS

### 2.1. Upper-rim modification of calix[n]arenes and their potential applications

**Figure 7:**
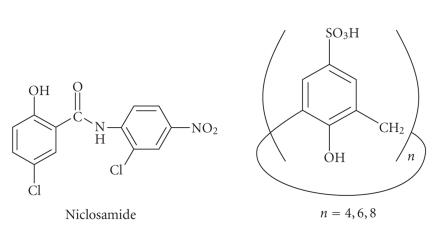
Molecular structure of niclosamide and 4-sulphanoto-calix[*n*]arenas.

Easy removal of the *t*-butyl group facilitates in the formation of diverse ranges of upper-rim functionalized calix[*n*]arenes. One of the most common features observed in almost every research work is the extended application of calixarene for the study of metal calixarene complexation behavior. Different substituents tend to influence the complexation behavior of the entire calixarene architecture. The bithiophene groups substituted at the upper rim gave rise to a host-guest complex of tungsten oxo calixarene complex [11]. Interaction of 4-sulfonic calix[*n*]arenes with niclosamide was investigated, which is a relatively new work in calixarenes-drug chemistry. The 4-sulfonic calix[8]arenes improved the solubility of the niclosamide the most, compared to -[4]arenes and -[6]arenes. The complex formed could be due to hydrogen bonding, hydrophobic bonding, and also possibly due to the electron donor-acceptor interactions [12] (Figure 7).

**Figure 8:**
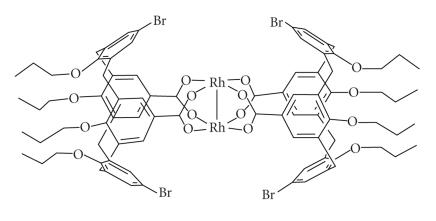
Bis(calix[4]arene) dirhodium complex.

Another 4-sulfonic calix[*n*]arene interaction with neutral molecule like furosemide has been observed. The molecular size of 4-sulfonic calix[6]arene influenced the increase in the solubility of the furosemide the most with the presence of noncovalent interaction behavior [13]. Napthalimido group introduced at the upper rim of calixarenes in varying proportions and a molecular capsule of two calixarenes is formed via perylene-bisimide spacer, the compound is used for UV and fluorescence studies [14]. An Rh–Rh unit formed an intermolecular link between two calix[4]arenes macrocycles and served as a ligand for transition metal catalysts [15] (Figure 8).

**Figure 9:**
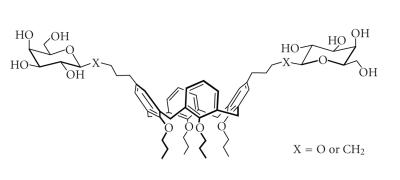
Calix[4]arene O- and C-Glycoconjugates.

Two pyridyl groups were linked via amide linkage at the upper rim and the calix[4]arene bispyridyl amides forming complexes with aromatic and alkyl dicarboxylic acids [16]. Diphenyl phosphino groups were attached at the upper rim of calix[4]arenes forming an organometallic ruthenium complex [17]. 4-hydroxybenzyl groups introduced at the upper rim of the calix[4]arene acted as anion binding groups [18]. Studies have revealed that the complexation of transition metals, like heavy metals, is most certainly favored by the incorporation of “softer” donor atoms such as nitrogen (as the amine), sulfur or phosphorus. A recent study of thiazolazo groups introduced at all the four positions of calix[4]arene was used to study for its recognition of heavy metal ions [19, 20]. Calix-O-glycosides were synthesized by multiple glycosylations of upper rim of calix[4]arene polyols [21]. These calyx sugars were successfully prepared by stereo selectively substituting at the upper rim with glycol (Figure 9).

**Figure 10:**
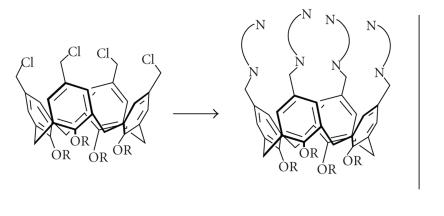
Calix[4]arenes linked to multiple bidentate N-donors.

Heterocyclylmethanamines attached at the upper rim of calix[4]arene (Figure 10) acted as a potential ligand for synthetic modeling of multinuclear metalloenzymes [22].

Semicarbazone was attached at two positions of the upper rim of calix[4]arene and fixed on a resin and studied for its sorption and separation studies of La(III), Ce(III), Th(IV), and U(VI) [23]. Isocyanide groups introduced at the upper rim of calix[4]arene was studied for its complexation with gold [24]. Adamantyl group introduced at the upper rim of calix[4]arenes gave rise to adamantyl calixarene [25].

### 2.2. Lower-rim modification of calix[n]arenes

The lower rim of calixarenes is less subjected to modification, but the applications of calixarenes substituted at the lower rim are far greater than substituted at the upper rim. Due to the expansion of the cavity after substitution, the lower rim can facilitate the complexation with bigger moieties like heavy metals and also organic molecules. The research put forth over here shows that after the structural modification of calix[*n*]arene, easy encapsulation of drugs, organic molecules as well as heavy metals is observed. Hence the phenolic hydroxyl groups at the lower rim of the calixarenes represent an excellent reactive function for the introduction of groups, which modify the shape, and the complexing properties of these molecules. Preliminary work on the lower rim of calixarenes has been started with alkylation and acylation reactions, which have been reviewed extensively in books [1, 9, 10] and review articles [5–8]. Different lower-rim modifications can be done as follows.

#### 2.2.1. Esterification

Esterification reactions on the lower rim of calix[*n*]arene have been the earliest reported work. The acylation and aroylation generally involve all the OH groups, a minor change in reaction conditions can change the outcome of the reaction. An excess or less amount of aroylating/acylating agent, the equivalents of reactants, the base, the solvent all contribute to the conformation of calix[*n*]arene (where *n* = 4/6). The main focus of aroylation has been with benzoyl chloride, *p*-nitro benzoyl chloride and 3,5-di nitro benzoyl chloride; although a number of other reagents are used for the synthesis of calixarene esters. Calixarene glycine ester acetamides have been synthesized from acetyl chlorides and glycine ethyl ester [26]. Esterification has also been reported for calix[8]arene where reaction of *p*-tert-butylcalix[8]arene with di-ethyl dibromomalonate gave the tetra ester derivative with a cone conformation [27]. O-acylated derivative of octa-tert-butyl calix[8]arene was prepared in high yields, by treating with dimethylacetamide in presence of acetic anhydride to give 95.4% octa-O-acetyl-octa(tert-butyl)calix[8]arene [28]. O-substituted calix[8]arenes were also prepared using allyl bromide in presence of pottassium hydroxide and polyethylene glycol di-ether in toluene to give its corresponding derivative [29].

#### 2.2.2. Etherification

**Figure 11:**
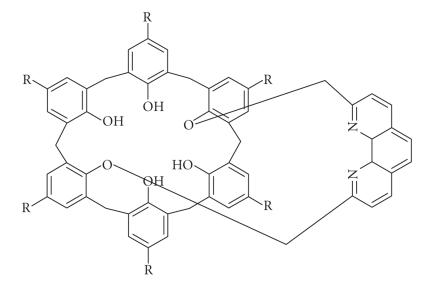
Calix[6]arene bridged by a 1,10-phenanthroline.

Alkylation has been studied in considerable detail in calix[4]arene series, and methods have been devised for preparing the mono, 1,2-di, 1,3-di, tri, and tetra ethers. Monoethers can be prepared in moderate-to-good yields by direct alkylation using an alkylating agent with sodium hydride as the base in toluene solution [30], barium hydroxide as the base in DMF solution [30] or 1.2 equivelent of a weak base and an excess of alkylating agent RX was used, where R includes methyl, ethyl, allyl or ethoxy carbonylmethyl [31]. Monobenzylation of 1,3-*p*-dinitrocalix[4]arene, with aluminum trichloride as the catalyst yielded its respective derivatives, the aroylation occurring preferentially on the aryl residues not containing the *p*-nitro groups [32]. It is observed that distal dialkylation leading to 1,3-diethers is generally much more easily achieved than proximal dialylation leading to 1,2-diethers. Under conditions similar to those leading to monoethers, but with an excess of the alkylating agent, 1,3-diethers were produced, often in very high yields; as, for example, 1,10-phenanthroline was used as a spacer to link intramolecularly and this derivative of -calix[6]arene was found to complex with Cu [33] (Figure 11).

Trimethylation of the parent calix[4]arene was accomplished with dimethylsulphate in DMF in the presence of BaO · Ba(OH)_2_ [10]. Higher yields of triether, however, can be obtained when the starting material is already partially alkylated. Another approach to the triether as well as the mono- and diethers involved protection-deprotection sequences [30]. Tetraalkylation of calix[4]arenes is generally carried out with an excess of the alkylating agent in the presence of the strong-base sodium hydride, although in some instances the much weaker-base potassium carbonate is also used.

**Figure 12:**
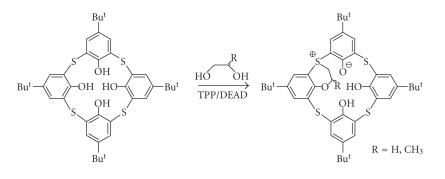
Bridged calix[4]arenas.

The first instance of alkylation of the sulfide bridge was achieved by the cyclocalkylation of *p*-tert-butylcalix[4]arene and *p*-tert-butylthiacalix[4]arene with various aliphatic glycols. This intrabridging of the calix[4]arene afforded 1,2- and 1,3-bridged calixarenes with O, S-cyclization, which caused the formation of sulfonium phenoxide betaines as shown in [34] (Figure 12).

New borono alkoxy calix[4]arenes were synthesized by alkylation, allylation, and hydroboration and this significantly gave rise to boronoalkoxycalix[4]arenes with 1,3-bridged structure which could coordinate significantly with monosaccarides [35]. *p*-tert-butylcalix[4]arene was reacted with *p*- and *m*-benzyl bromides in the presence of alkali metal carbonates, and silylation of these derivatives gave their respective derivatives [36]. A series of six calix[4]arene derivatives bearing allyl groups and/or benzyl groups have been functionalized at the phenolic oxygen atoms [37]. *p*-*t*-butylcalix[4]arene diamides were synthesized in a stepwise method where it was first reacted with ethylbromoacetate, then the hydrolysis was followed by the conversion to acid chloride and amidation [38].

**Figure 13:**
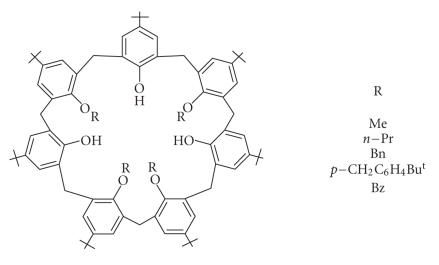
Selectively functionalized calix[7]arenas.

Regioselective synthesis of monoalkylethers of *p*-tert-butyl calix[6]arene in good yield was achieved with a variety of electrophiles using 2,2 equivelent, of pottassium carbonate as base in acetonitrile under ultrasonic irradiation at ambient temperature [39]. The first example of selectively functionalized calix[7]arenes has been obtained by weak-base promoted O-alkylation or O-benzoylation of *p*-tert-butylcalix[7]arene. Mono, 1,3- and 1,4-disubstituted calix[7]arenes have been obtained in workable yields, while the 1,2,4,6-tetra substitution was achieved with surprisingly high selectivity by 50–88% yield by using pottassium carbonate as the base [40] (Figure 13).

The calix[8]arenes present an interesting case where in addition to the fully O-substituted calix[8]arenes, which was obtained by treatment with strong bases and a large excess of derivatizing agent [41], twenty eight partially alkylated calix[8]arenes were also obtained. Neri and coworkers have reported the first success in selective lower-rim substitution and provided details for the preparation of 1,3,4,6-tetra-O-arylmethyl ether of tert-butyl-calix[8]arene obtainable in yields of 20–41% using potassium carbonate as the base [42]. The direct methylation was studied in considerable detail, and procedures were worked out for generating some of the partially methylated compounds in isolable yields [43].

**Figure 14:**
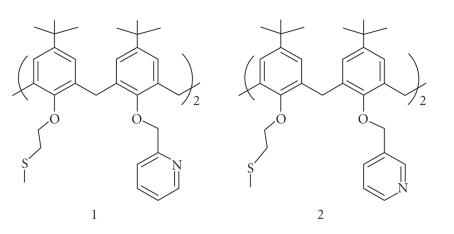
Lower-rim calix[4]arene derivatives with mixed pendent arms.

Alkylation with aryl methyl halides containing hetero atoms provided still another route for the introduction of functional groups onto the lower rim [44]. Methylthioethoxy and pyridyl 2 methyl oxy groups were introduced at the lower rim of calix[4]arene to give rise to bis derivatives of both types 1 and 2 [45] (Figure 14).

**Figure 15:**
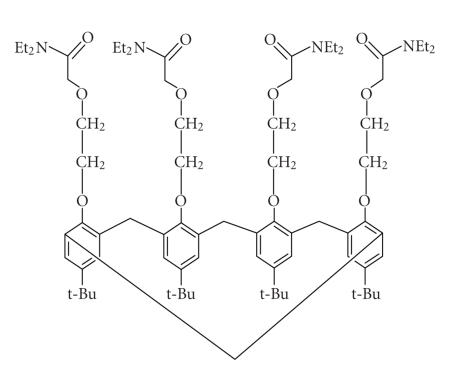
2-diethylcarbamoylmethoxyethoxy substituted *t*-butyl-calix[4]arene.

Polysiloxane derivatives of calix[4]arene were prepared in presence of chloroplatinic acid [46]. Proximal O,O′ capped calix[4]arenes with a disiloxane bridge is synthesized using cesium carbonate as the base in THF, also providing an alternative of desilylation and thereafter alkylation using benzyl bromide with potassium tert-butoxide as a base to give rise to unsymmetrical calix[4]arenas [47]. An ether-amide linkage was synthesized using 2-diethylcarbamoylmethoxyethoxy group at the lower rim of *t*-butyl-calix[4]arene [48] (Figure 15).

**Figure 16:**
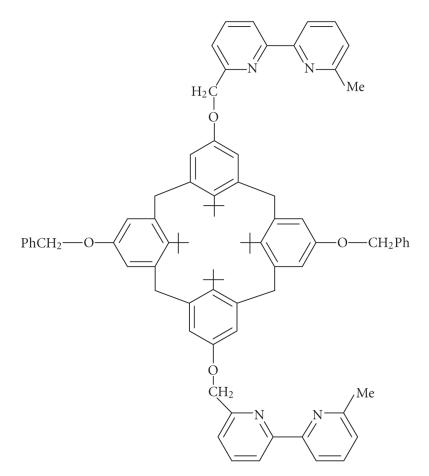
Calix[4]arene-based bipyridine podand.

Calix[4]arene containing pyridinyl moiety was synthesized forming a tetraether derivative [49]. Selective 2,2′-bpyridine units at 1,3 position and two benzyl units at 2,5 positions at the lower rim of calix[4]arene was prepared [50] as in (Figure 16).

A new family of calix[4]arene was prepared by the incorporation of 2,2′-bithiazole units [51]. Water soluble calix[4]arene incorporating both sulfonate groups at its upper rim and 2,2′-bipyridine groups at its lower rim was also prepared [52]. Etherification in 1,3-alternate conformation was carried out on calix[4]arene using R = 2-MeOC_6_H_4_OCH_2_CH_2_ with considerable good yields [53].

Schiff-base derivatives were synthesized using the amino ethoxy derivatives of calix[4]arene with the aromatic aldehydes in high yields [54]. Copolyethers and polyurethanes containing lower- and upper-rim calix[4]arene units in the fixed cone conformation were prepared by reacting the bisphenol with the distal calix[4]arene diols [55].

#### 2.2.3. Bridged calix[*n*]arenes

The first synthesized lower-rim 1,3 ring bridged calixarene is the calix[4]arenecrown [56] in early 1980s and now represented by dozens of examples [1, 6, 9]. The parent calixcrowns as well as their dimethyl ethers retain some conformational flexibility and can exist in cone, partial cone, and 1,3-alternate conformations, but ethers with larger groups such as isopropyl and benzyl have fixed cone conformations. There is also the report of the dialkylation of calix[4]arene capped by diamide bridges which gave rise to fully substituted compounds of 1,3-alternate conformation The diesters were then cyclized with diamines to afford doubly capped derivatives [57]. Other crown ether-type bridges have been synthesized [54] like the aza crowns [58], the bipyridyls [58], and a variety of aza crown-type structures. Another type of 1,3-bridged calix[4]arene including those with a double bond in the bridge have been prepared by the ruthenium-catalyzed coupling of the 1,3-bisbutenyl ether [59]. Only a few examples of calixcrowns of the larger calixarenes have been reported. New borono alkoxy calix[4]arenes were synthesized by alkylation, allylation, hydroboration, and significantly gave rise to boronoalkoxycalix[4]arenes with 1,3-bridged structure which could coordinate significantly with monosaccarides [60]. Calix[4]arene dibenzo crown ether has been prepared in 1,3-alternate conformation using R = C1–C40 *n*-alkyl chains [61].

**Figure 17:**
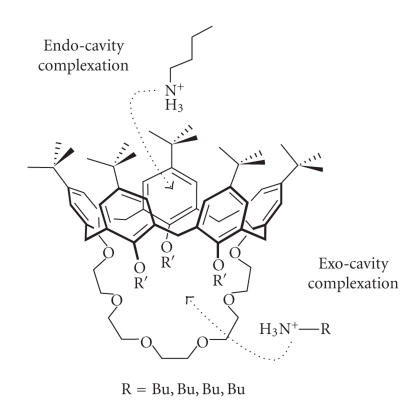
1,3-bridged calix[5]arene crown-6 ethers.

Azo benzene derivatized crown *p*-tert-butyl calix[4]arene was synthesized in a stepwise method and a 1,3-bridged structure was formed [62]. Crown ethers derived from bicyclocalix[4]arenes in which the opposite phenolic units are connected by a poly oxyethylene bridge at the lower rim and a 2,6-di methyllene-4-nitrophenol bridge at the upper rim were reported [63]. These derivatives were found to be potential ligands for the complexation of potassium and cesium. Calix[4]arene derivative containing pyridyl methoxy group at its lower rim has been synthesized using potassium carbonate and sodium iodide [64]. The spanning of *t*-Bu-calix[5]arene has been accomplished with tetraethyleneyethoxy and pentaethyleneoxy chains joining the 1,3 rings and with hexaethyleneoxy chains joining the 1,3 rings [65]. Tri-O-substituted 1,3-bridged calix[5]arene-crown-6ethers bearing alkyl, arylalkyl, alkoxyalkyl, and alkoxycarbonylmethyl residues were attached at the lower rim of the calix[5]arene with cone conformations although they possess a bulky structure [66] (Figure 17).

**Figure 18:**
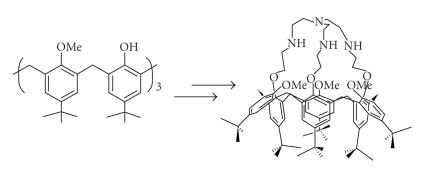
First *C*_3*v*_-symmetrical calix[6](aza)crown.

The first *C*_3*v*_-symmetrical calix[6](aza)crown has been obtained in five-step synthesis procedure to give a cone conformation and prevent a ring inversion [67] (Figure 18). Diester intrabridging of *p*-tert-butyl calix[8]arene was afforded using spanner adipoyl chloride in the presence of sodium hydride as the base and hence yielded singly and doubly intrabridged esters; xantheno calix[8]arenes were also obtained in the course of the rearrangement of the intermediate product [68].

**Figure 19:**
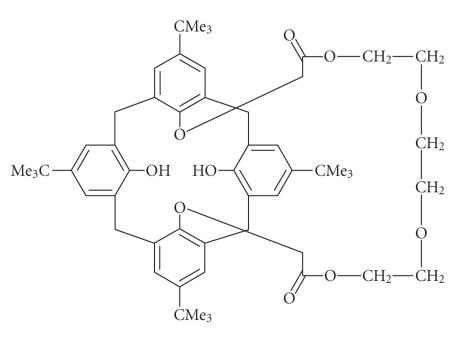
Lower-rim functionalized acetate derivative of -calix[4]arene.

Examples of porphyrins quadruply attached to the lower rim of calixarene have been reported [69]. *p*-tert-butyl calix[4]arenes with diester bridge spanning the 1,3-distal positions on the lower rim were prepared by cyclocondensation of polyethylene glycol bis bromo acetates with ter-butylcalix[4]arene [70] (Figure 19).

One of the few lower-rim-spanned calix[5]arenes that has been reported is the 1,3-di-ester obtained in low yield from *p*-tert-butyl calix[5]arene and *o*-pthaloyl chloride [71] but considerable attention has been devoted to bridge the lower rim of calix[6]arenes with spanners other than polyethyleneoxy. An *m*-xylene bridged calix[6]arene has been synthesized with the other positions being functionalized by methoxy groups [72]. It has been observed that for big spanners, the calix[6]arene is conformationally inflexible and retains its cone structure.

**Figure 20:**
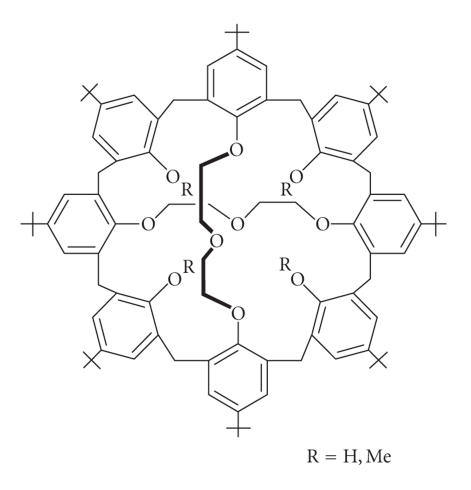
D_2d_-symmetrical 1,5-3,7-calix[8]bis-crown-3 derivatives.

Calix[8]arenes have been quadruply-spanned by a durylene moiety to give a structure that has fixed pseudopleated loop conformation [70], a potential moiety for the complexation of different metal ions [73]. Biscrowned calix[8]arenes were synthesized by alkylating *p*-tert-butylcalix[8]arene or calix-[8]monocrowns with diethylene glycol tosylate [74] (Figure 20) triethylene glycol ditosylate, in the presence of various bases where 22 possible isomers were isolated in varying yields.

**Figure 21:**
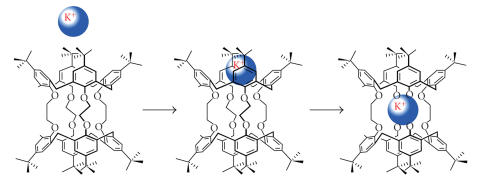
Calix[4]tubes.

Bis-calixarenes connected by four tetraalkyltetra(tosyl-oxyethoxy) groups have been reported called as calixtubes, in high yields in 71% yield. These molecules proved highly selective for complexation of potassium over other I-group cations and barium [75] (Figure 21). Calix[4]crown diacylamides with two acetaminoanthraquinone units at the lower rim have been reported [76].

## 3. APPLICATIONS

The spurt in the increase of literature regarding calixarenes in the last 25 years can be ascribed to the growing interest in introducing different functional groups via different synthetic procedures. But also the major factor that has contributed for the proliferation of these research papers is the tailor-made structure of calixarenes for its use as complexing agents, for it is this possibility and its potential that has brought for calixarenes this recognition that they enjoy today. The use of these modified calixarenes as sensors for metal ion, organic/neutral molecules, and drugs recognition has brought calixarenes to limelight. They have become a wonder molecule at the hands of a chemist. This wonder molecule has its roots in host-guest chemistry.

During the molecular evolution of biological system, the highly selective complexation process between the host and the guest must have played a central role; this attribute of biological life was mimicked in synthetic chemistry which later came to be known as the host-guest chemistry. A molecular complex is composed of at least one host and one guest components. The host is an organic molecule or ion whose binding sites converge. The guest is an organic molecule or ion or metal ion whose binding sites diverge [77]. The complexes of the host-guest chemistry are held together in unique structural relationship by forces other than those of covalent nature. They can be pole-pole, pole-dipole or dipole-dipole variety, more specifically, the components of complex are bound together by hydrogen bond, by ion-pair, by *π*-*π* stacking interactions, and by van-der Waals forces [78, 79]. There are a number of ways by which the complexation phenomena could be studied. In addition to the powerful spectrophotometric methods now available, most often NMR and/or UV-Vis spectrometry and various other techniques like mass spectrometry are also being used [80]. The precise structures of complexes are most directly obtained by X-ray crystallography, and the reasonable assumption is generally made that the solid structure architecture is similar to the solution state. The ability in terms of sensitivity and the selectivity of the calixarene as a host to discriminate among a group of guests makes it a special class of subject in supramolecular chemistry.

### 3.1. Lower-rim esters

Much more effective than the simple ethers, the esters have been extensively studied. The earliest to be studied among this family of compounds were esters for which it was determined, using phase transfer extraction measurements, that the cyclic tetramers, pentamers, and hexamers extract all of the alkali cations, the cyclic tetramer works best with Na^+^, the cyclic pentamer better with K^+^, Rb^+^, and Cs^+^, and the cyclic hexamer best with Rb^+^ and Cs^+^ but very poorly with Na^+^, and the cyclic heptamer and octamer are quite ineffective [81].

McKervey, Diamond, and Svehla continued to publish on the use of calixarenes esters as electrochemical sensors. The calix[4]arene tetra ethyl ester synthesized by this group continues to be commercially available as sodium selective electrode and is commonly used in hospitals for measuring sodium in blood. This group has also showed the calixarenes could produce electrodes selective for potassium and cesium [6]. Ester derivatives, the synthesis of which can be achieved smoothly, binds amines, with a preference for shorter amines [82].

The ligand discriminates according to guest hydrophobicity and shows selectivity for phenylalanine and tyrosine esters over glycine, alanine and 4-aminobutyric acid, with the interaction primarily taking place due to tripodal hydrogen bonding. Ammonium and alkylammonium ions can be sensed by calixarenes where diquinone is part of the macrocycle together with ligating sites such as ester or amide for hydrogen bonding [83, 84].

### 3.2. Lower-rim ethers, ketones, amides, and carboxylic acids

**Figure 22:**
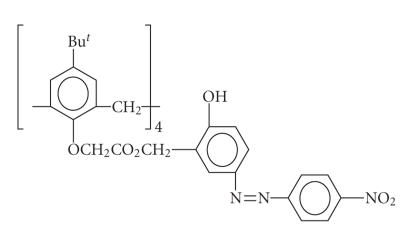
Chromogenic nitrophenylazophenol calix[4]arene.

Lower-rim ethers and their counterparts, ketones, amides, and carboxylic acids have good complexing properties. The earliest examples of lower-rim-substituted calixarenes investigated for their complexation properties are the ethyleneoxy compounds [85, 86] which show only a modest degree of cation binding agency. As it is the case for crown ethers, calixarenes with oxygen donor atoms turned out to be suitable for selectively binding alkali ions. The ligands are more hydrophobic compared with crown ethers and the membranes therefore are more stable. Nitrophenol or azophenol moieties on calix[4]arenes equipped with additional ester groups [87] transform the Li/Na selectivity in organic solutions into a bathochromic shift from 350 to 425 nm with the help of an auxillary base to support the deprotonation. The calixarene with a nitrophenylazophenyl group is rather versatile. It not only detects lithium ions in the presence of weak base, but also in turn detects weak bases such as volatile amines when Li^+^ is already present in the membrane [88, 89] (Figure 22).

Ketones have complexing features [90] similar to those of the esters previously described. Stability constant measurements and extraction data indicate that the cyclic tetrameric ketones are better than their ester analogs for the extraction of Li^+^ and also for Rb^+^ and Cs^+^. The ketone has a broader range of extraction capability than its cyclic tetramer and hexamer counterparts but shows little selectivity among the cations.

**Figure 23:**
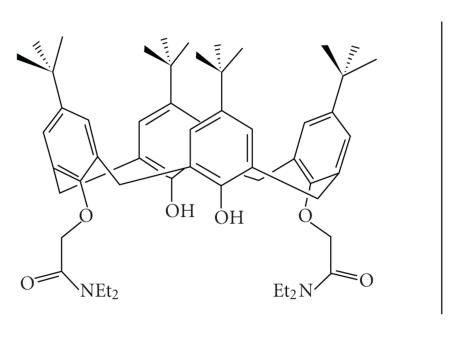
1,3-bis(diethyl amide)-substituted calix[4]arene.

Amides were first prepared and studied by Ungaro et al. [91] and subsequently in considerable detail by Mckervey et al. [6, 92] and Beer et al. [93–96] (Figure 23). As against the ethers, esters, and ketones, the amides very effectively complex alkaline earth cations [91]. In extraction studies the cyclic tetramers are shown to prefer Eu^+3^ over Pr^+3^ and Yb^+3^, with Eu^+3^ the cyclic hexamers being better than the cyclic tetramers showing the highest extraction effectiveness [97].

Trivalent cations are also effectively bound by the amides [98] like Pr^+3^, Eu^+3^, and Yb^+3^. The amides prove to be superior to the esters and ketones for complexing Ag^+^ [91] and within the amide series the cyclic pentamer is an especially strong complexing agent. The larger Tl^+^ cation forms weaker complexes than Ag^+^. Among the ester, acid, or amide groups anchored to calixarenes, the latter turned out to form most stable complexes with alkaline earth due to the high carbonyl group polarity [99].

With phoshphine oxide groups appended to the lower rim, in good Ca^+2^/ Na^+^ selectivity and membrane durability is achieved in ISEs. Expanding the calix[*n*]arene cavity from *n* = 4 over 5 to 6 changes the Sr/Na selectivity of the amide derivatives from 0.09 to 2.8 to 760 in extraction which further improves with alkyl ether instead of *t*-butyl groups in 4-position of the phenyl groups [100]. Amide derivatives of calixarenes have also been used for Sr^+2^ separation in synergistic mixtures with hydrophobic anion [98, 101] which should work in ISE membranes as well.

**Figure 24:**
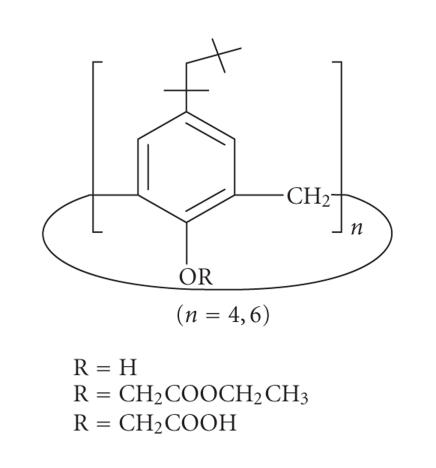
Calixarene esters for complexation with rare earths.

Carboxylic acids differ from the esters, ketones, and amides in having ionizable groups. The carboxylic acids are all more effective complexing agents for alkali cations than the corresponding esters, ketones, and amides. Although the calixarene carboxylic acids form complexes with some of the alkali metal cations, [102] the fact that they have an even greater capacity for alkaline earth cations was first realized in the mid 1980s [86] and subsequently studied in some detail. The acids form stronger complexes with lanthanide cations like Pr^+3^, Eu^+3^, and Yb^+3^ than with alkali and alkaline earth cations. The calix[4]arene dicarboxylic acid and calix[6]arene ester are useful for the extraction of the rare earths [103, 104] (Figure 24).

Some of the carboxylic acids have proved to be highly effective complexation agents for the uranyl cation [105]. The analogous *p*-tert-butylcalix[5]arene and -[6]arenes are somewhat less effective uranophiles, but the corresponding hydroxamic acids (*n* = 6) is even better and also competes with CO_3_^−2^ ions [106–108].

### 3.3. Lower-rim nitrogen, sulfur, and phosphorus containing groups

**Figure 25:**
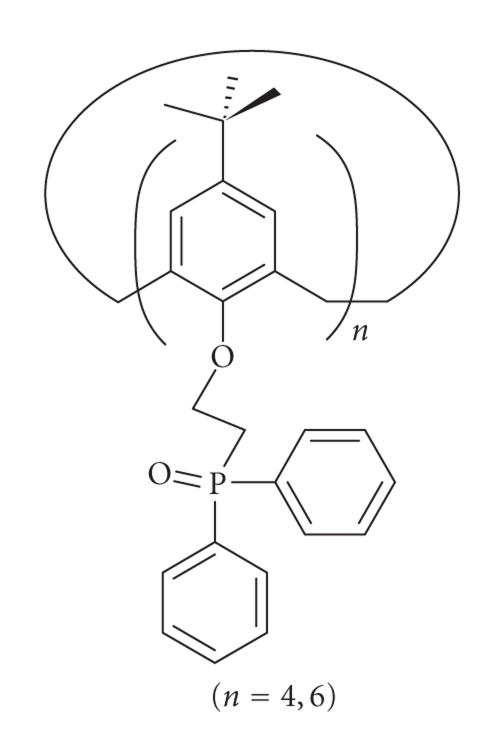
Phosphine oxide derivatives of calix[6]arene.

The McKervey group introduced tetrameric calixarenes where nitrogen and sulfur were used in the coordination center and they were found to be selective for soft heavy metal ions such as silver [6]. Electrodes based on the hexamer derivative of the phoshphine oxide series of derivatives were found to have excellent selectivity for lead ions [6] (Figure 25).

**Figure 26:**
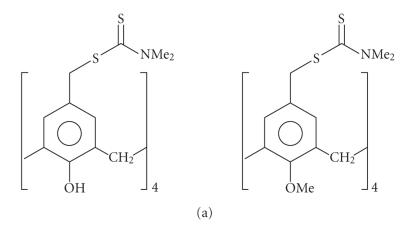

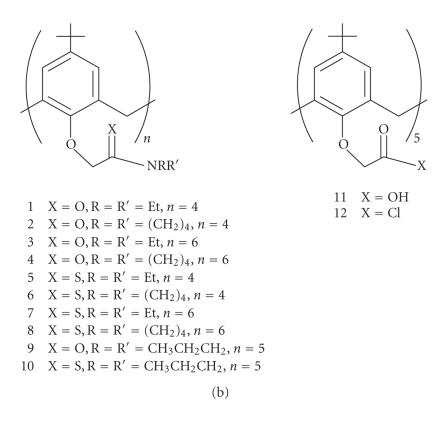
(a) Thia calix[*n*]arenas; (b) thia calix[*n*]arenas.

Thioester groups in calix[4]arenes lower the selectivity over sodium ions to some extent due to their carbonyl oxygens. Thiocarbamoyl groups or dithiocarbamoyl groups attached to the calixarene skeleton (Figures 26(a) and 26(b)) provide selectivity for Ag^+^, Pd^+2^, Hg^+2^ and other soft metal ions over hard ones [109, 110].

A class of hosts forms only weak complexes with the alkali and alkaline earth cations but forms strong complexes with Ag^+^, Pb^+2^, and Cd^+2^ being the thioamides [110]. The cyclic pentamer is particularly effective for the extraction of Cd^+2^ and the cyclic hexamer shows a high affinity towards Ag^+^/ Cu^+2^ and Ag^+^/ Pb^+2^ selectivities. Calixarenes binding sites at the sulfur atoms have the potential for forming ditopic bimetallic complexes. There is some evidence that this has been accomplished with Ag^+^ and Na^+^ for the compound in which *n* = 6 although the same compound excludes the Na^+^ ion [111]. Calixarenes containing diphenylphosphoryl acetamide moieties on the upper rim [112] and lower rim [113], respectively, are highly efficient extractants for Eu^+3^, Th^+3^, Np^+3^, Pu^+3^, and Am^+3^. The N,N-dimethyl dithiocarbamoyl ethyl ether is an effective extractant [114, 115] for Pd^+2^ and other heavy metals.

### 3.4. Lower-rim bridged calixarenes

Crowned calixarenes called calixcrowns, carrying bridging polyethylenoxy moieties on the lower rim and being first prepared by Ungaro et al. [56] in 1983, have proved to be very effective cation-complexing agents. They are characterized by a high degree of molecular preorganization and therefore achieve even higher selectivity, especially among alkali ions. In contrast to the esters, ketones, and amides, which are selective for Na^+^, the calixcrowns show a preference for the larger cations. Several calixcrown dialkyl ethers have been studied [28, 68]. The crown moiety and the calixarene restrict each other's molecular flexibility leading to a better discrimination by ion size. The ligand [116] with a narrow cavity for maximum Na/K selectivity, which when mixed with flurophore makes an optode, can be miniaturized for intracellular measurements.

**Figure 27:**
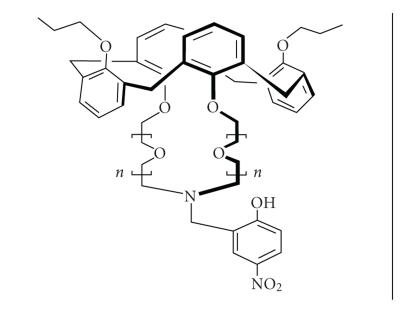
Calix[4]azacrown ethers in the 1,3-alternate conformation.

An alternate conformation, but with azacrown-5 moieties and its monocrown relatives, shows K^+^ selectivity in transport as well as in ISEs which translates into an optical signal with the help of the nitrophenol chromophoric group [117–119] (Figure 27).

**Figure 28:**
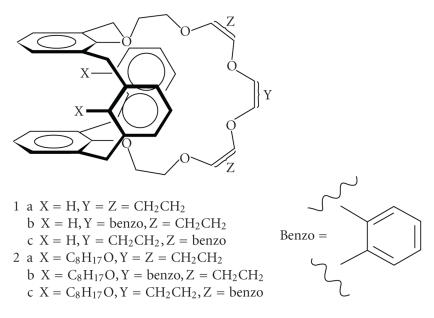
Doubly crowned calix[4]arene.

Attaching benzo or naptho groups to the crown moiety increases the Cs/Na selectivity. The Cs/K selectivity on the other hand improves to over 4000 after removal of the two phenolic oxygens outside the crown cavity [120]. Doubly crowned calix[4]arene such as (Figure 28) avoid the need for protective groups during synthesis and can bind two metal ions in one molecule.

## 4. CONCLUDING REMARKS

Since last few decades calix[*n*]arenas (*n* = 4–8), like cyclodextrins and crown ethers, have played an important role as host for ions, neutral molecules, and organic guests. The first part of the review mentions in detail the “calixarenes,” their origin, and the different modifications that are possible within this molecule. The calixarene molecule due to its reactive positions at the upper rim (removal of alkyl group) and the lower rim (removal of hydroxyl group) can be functionalized with identical or different binding groups. The few examples discussed here demonstrate the unlimited possibilities that exist for molecules like calixarenes for modifications. Their modified potential applications have been discussed towards the second half of the article, which range from their use as selective sensors for different analytical applications and medical diagnostics to their use in decontamination of wastewater, construction of electrodes, and membranes for transportation.

## References

[1] Lehn JM (1995). *Supramolecular Chemistry*.

[2] Pedersen CJ (1967). Cyclic polyethers and their complexes with metal salts. *Journal of the American Chemical Society*.

[3] Shinkai S (1993). Calixarenes—the third generation of supramolecules. *Tetrahedron*.

[4] Gutsche CD, Dhawan B, No KH, Muthukrishnan R (1981). Calixarenes. 4. The synthesis, characterization, and properties of the calixarenes from *p-tert*-butylphenol. *Journal of the American Chemical Society*.

[5] Ikeda A, Shinkai S (1997). Novel cavity design using calix[*n*]arene skeletons: toward molecular recognition and metal binding. *Chemical Reviews*.

[6] McMahon G, O'Malley S, Nolan K, Diamond D (2003). Important calixarene derivatives—their synthesis and applications. *Arkivoc*.

[7] Sliwa W (2002). Calixarene complexes with transition metal, lanthanide and actinide ions. *Croatica Chemica Acta*.

[8] Menon S, Gidwani MS, Agrawal YK (2003). Chromogenic calixarenes. *Reviews in Analytical Chemistry*.

[9] Böhmer V, Shivanyuk A, Mandolini L, Ungaro R (2000). Calixarenes in self-assembly phenomena. *Calixarenes in Action*.

[10] Gutsche CD, Stoddart J (1989). Calixarenes. *Monographs in Supramolecular Chemistry*.

[11] Vigalok A, Swager TM (2002). Conducting polymers of tungsten(VI)-oxo calixarene: intercalation of neutral organic guests. *Advanced Materials*.

[12] Yang W, de Villiers MM (2004). The solubilization of the poorly water soluble drug nifedipine by water soluble 4-sulphonic calix[*n*]arenes. *European Journal of Pharmaceutics and Biopharmaceutics*.

[13] Yang W, de Villiers MM (2004). Aqueous solubilization of furosemide by supramolecular complexation with 4-sulphonic calix[*n*]arenes. *Journal of Pharmacy and Pharmacology*.

[14] Vysotsky MO, Böhmer V, Würthner F, You C-C, Rissanen K (2002). Calix[4]arene-functionalized naphthalene and perylene imide dyes. *Organic Letters*.

[15] Seitz J, Maas G (2002). Calixarenes as ligands for transition-metal catalysts: a bis(calix[4]arene-11,23-dicarboxylato) dirhodium complex. *Chemical Communications*.

[16] Miyaji H, Dudic M, Tucker JHR (2002). Bis(amidopyridine)-linked calix[4]arenes: a novel type of receptor for dicarboxylic acids. *Tetrahedron Letters*.

[17] Lejeune M, Jeunesse C, Matt D, Kyritsakas N, Welter R, Kintzinger J-P (2002). Positioning of transition metal centres at the upper rim of cone-shaped calix[4]arenes. Filling the basket with an organometallic ruthenium unit. *Journal of the Chemical Society, Dalton Transactions*.

[18] Arduini A, Giorgi G, Pochini A, Secchi A, Ugozzoli F (2001). Anion allosteric effect in the recognition of tetramethylammonium salts by calix[4]arene cone conformers. *Journal of Organic Chemistry*.

[19] Akdoǧan A, Deniz M, Cebecioǧlu S, Şen A, Deligöz H (2002). Liquid-liquid extraction of transition metal cations by nine new azo derivatives calix[*n*]arene. *Separation Science and Technology*.

[20] Lu J-Q, He X-W (2002). *Gaodeng Xuexiao Huaxue Xuebao*.

[21] Dondoni A, Kleban M, Hu X, Marra A, Banks HD (2002). Glycoside-clustering round calixarenes toward the development of multivalent carbohydrate ligands. Synthesis and conformational analysis of calix[4]arene *O*- and *C*-glycoconjugates. *Journal of Organic Chemistry*.

[22] Spencer DJE, Johnson BJ, Johnson BJ, Tolman WB (2002). Calix[4]arenes linked to multiple bidentate N-donors: potential ligands for synthetic modeling of multinuclear metalloenzymes. *Organic Letters*.

[23] Jain VK, Handa A, Pandya R, Shrivastav P, Agrawal YK (2002). Polymer supported calix[4]arene-semicarbazone derivative for separation and preconcentration of La(III), Ce(III), Th(IV) and U(VI). *Reactive and Functional Polymers*.

[24] Gagnon J, Drouin M, Harvey PD (2001). Upper-rim functionalization of calix[4]arene by chloro(isocyanide)gold(I) groups: an entry to polymetallic architecture. *Inorganic Chemistry*.

[25] Shokova EA, Khomich AN, Kovalev VV (2001). Selective upper-rim adamantylation of calix[4]arenes. *Russian Journal of Organic Chemistry*.

[26] König B, Fricke T, Gloe K, Chartroux C (1999). Synthesis and metal-ion extraction properties of *para-tert*-butylcalixareneglycine ester acetamides. *European Journal of Inorganic Chemistry*.

[27] Yuan DQ, Zhu WX, Wang RJ, Zhao MX, Yan X, Zheng QY (2002). Synthesis and crystal structure of a novel calix[8]arene ester derivative. *Chinese Chemical Letters*.

[28] Yamanaka S, Sugata K

[29] Yamanaka S, Sugata K

[30] Iwamoto K, Araki K, Shinkai S (1991). Syntheses of all possible conformational isomers of O-alkyl-p-t-butylcalix[4]arenes. *Tetrahedron*.

[31] Groenen LC, Ruel BHM, Casnati A (1991). Synthesis of monoalkylated calix[4]arenes via direct alkylation. *Tetrahedron*.

[32] Sharma SK, Gutsche CD (1996). Selective lower rim reactions of 5,17-upper rim-disubstituted calix[4]arenes. *Journal of Organic Chemistry*.

[33] Ross H, Lüning U (1997). Concave reagents—23. Synthesis of calix[6]arene bridged by a 1,l0-phenanthroline. *Tetrahedron Letters*.

[34] Csokai V, Grün A, Balázs B, Tóth G, Horváth G, Bitter I (2004). Unprecedented cyclizations of calix[4]arenes with glycols under the mitsunobu protocol—part 2.^1^ O,O-and O,S-bridged calixarenes. *Organic Letters*.

[35] Lu K, Wu Y-J, Xing Y-J, Zou D-P, Zhou Z-X (1999). Studies on the syntheses and properties of boronoalkoxycalix[4]arenes. *Chinese Journal of Chemistry*.

[36] Alekseeva EA, Mazepa AV, Gren' AI (2001). Synthesis and conformational characteristics of benzyl-substituted *p-tert*-butylcalixarenes. *Russian Journal of General Chemistry*.

[37] Daniel C, Mauro M, Chris R, Chitosi K, Akio Y, Mikio O (1999). *Australian Journal of Chemistry*.

[38] Yu T-X, Yang X-J, Xiao Y-J, He Y-B (2001). The synthesis of a few p-t-butycalix [4] arene diamides and its complexation with some ions. *Wuhan Daxue Xuebao*.

[39] Semwal A, Bhattacharya A, Nayak SK (2002). Ultrasound mediated selective monoalkylation of 4-tert-butylcalix[6]arene at the lower rim. *Tetrahedron*.

[40] Martino M, Gregoli L, Gaeta C, Neri P (2002). Regioselective *O*-substitution of *p-tert*-butylcalix[7]arene. *Organic Letters*.

[41] Neri P, Battocolo E, Cunsolo F, Geraci C, Piattelli M (1994). Study on the alkylation of *p-tert*-butylcalix[8]arene. Partially *O*-alkylated calix[8]arenes. *Journal of Organic Chemistry*.

[42] Neri P, Geraci C, Piattelli M (1993). Tetra-*O*-benzylated calix[8]arenes with *C*
_4_ symmetry. *Tetrahedron Letters*.

[43] Neri P, Consoli GML, Cunsolo F, Rocco C, Piattelli M (1997). Alkylation products of a calix[8]arene trianion. Effect of charge redistribution in intermediates. *Journal of Organic Chemistry*.

[44] Honeychurch KC, Hart JP, Cowell DC, Arrigan DWM (2002). Voltammetric behavior and trace determination of cadmium at a calixarene modified screen-printed carbon electrode. *Electroanalysis*.

[45] Danil de Namor AF, Al Rawi N, Piro OE, Castellano EE, Gil E (2002). New lower rim calix(4)arene derivatives with mixed pendent arms and their complexation properties for alkali-metal cations. Structural, electrochemical, and thermodynamic characterization. *Journal of Physical Chemistry B*.

[46] Shimizu S, Sasaki Y, Okazaki J

[47] Narumi F, Morohashi N, Matsumura N, Iki N, Kameyama H, Miyano S (2002). Proximal *O, O′*-capped calix[4]arenes with a disiloxane bridge as highly efficient synthetic intermediates for 1,2-dialkylation at the lower rim. *Tetrahedron Letters*.

[48] Ramírez FD-M, Charbonnière L, Muller G, Scopelliti R, Bünzli J-CG (2001). A *p-tert*-butylcalix[4]arene functionalised at its lower rim with ether-amide pendant arms acts as an inorganic-organic receptor: structural and photophysical properties of its lanthanide complexes. *Journal of the Chemical Society, Dalton Transactions*.

[49] Han B, Liu Y, Chen Z, Chen R (2000). *Huaxue Tongbao*.

[50] Dalbavie J-O, Regnouf-de-Vains J-B, Lamartine R, Perrin M, Lecocq S, Fenet B (2002). A calix[4]arene-based bipyridine podand as versatile ligand for transition metal cations. *European Journal of Inorganic Chemistry*.

[51] Oueslati F, Dumazet-Bonnamour I, Lamartine R (2001). Synthesis of new chromogenic 2,2′-bithiazoylcalix[4]arenes. *Tetrahedron Letters*.

[52] Psychogios N, Regnouf-de-Vains J-B (2002). A new water-soluble calix[4]arene podand incorporating
*p*-sulphonate groups and 2,2′-bipyridine chelating units. *Tetrahedron Letters*.

[53] Xing Y-J, Zhou Z-X, Wu Y-J (2001). Syntheses and properties of new pendant-armed calix[4]arene derivatives as cesium selective ionophore. *Chinese Journal of Chemistry*.

[54] Liu Y, Zhao B-T, Wang H, Chen Q-F, Zhang H-Y (2001). *Chinese Journal of Chemistry*.

[55] Dondoni A, Marra A, Rossi M, Scoponi M (2004). Synthesis and characterization of calix[4]arene-based copolyethers and polyurethanes. Ionophoric properties and extraction abilities towards metal cations of polymeric calix[4]arene urethanes. *Polymer*.

[56] Wieser C, Matt D, Fischer J, Harriman A (1997). Capping calixarenes with metallodiphosphine fragments: towards intracavity reactions. *Journal of the Chemical Society, Dalton Transactions*.

[57] Bitter I, Grün A, Balázs B, Tóth G, Horváth G, Töke L (1999). Studies on calix(aza)crowns. III. Synthesis of 1,3-alternate calix[4]arenes capped by diamide bridges. *Synthetic Communications*.

[58] Liu Y, Zhao B-T, Huang G, Zhang H-Y, Wang H (2001). An uncommon calix[4]azacrown-4 dimer assembled by hydrogen-bonded interaction. *Journal of Chemical Research*.

[59] Tuntulani T, Ruangpornvisuti V, Tantikunwatthana N (1997). Synthesis of the tripodal-amine capped benzo crown *p-tert*-butylcalix[4]arene and its host-guest chemistry. *Tetrahedron Letters*.

[60] Lu K, Wu Y-J, Xing Y-J, Zou D-P, Zhou Z-X (1999). Studies on the syntheses and properties of boronoalkoxycalix[4]arenes. *Chinese Journal of Chemistry*.

[61] Oh W-Z, Lee KW, Choi WK

[62] Pipoosananakaton B, Sukwattanasinitt M, Jaiboon N, Chaichit N, Tuntulani T (2000). New azobenzene crown *p-tert*-butylcalix[4]arenes as switchable receptors for Na^+^ and K^+^ ions: synthesis and isomerization studies. *Bulletin of the Korean Chemical Society*.

[63] Wąsikiewicz W, Ślaski M, Rokicki G, Böhmer V, Schmidt C, Paulus EF (2001). Crown ethers derived from bicyclocalix[4]arenes as chromoionophores. *New Journal of Chemistry*.

[64] Tuntulani T, Tumcharern G, Ruangpornvisuti V (2001). Recognition studies of a pyridine-pendant calix[4]arene with neutral molecules: effects of non-covalent interactions on supramolecular structures and stabilities. *Journal of Inclusion Phenomena and Macrocyclic Chemistry*.

[65] Gale PA, Sessler JL, Lynch V, Sansom PI (1996). Synthesis of a new cylindrical calix[4]arene-calix[4]pyrrole pseudo dimer. *Tetrahedron Letters*.

[66] De Salvo G, Gattuso G, Notti A, Parisi MF, Pappalardo S (2002). Shape recognition of alkylammonium ions by 1,3-bridged calix[5]arene crown-6 ethers: *endo*- vs *exo*-cavity complexation. *Journal of Organic Chemistry*.

[67] Jabin I, Reinaud O (2003). First *C_3v_*-symmetrical calix[6](aza)crown. *Journal of Organic Chemistry*.

[68] Consoli GML, Geraci C, Cunsolo F, Neri P (2003). Diester intrabridging of *p-tert*-butylcalix[8]arene and unexpected formation of the monospirodienone derivative. *Tetrahedron Letters*.

[69] Gale PA, Sessler JL, Lynch V, Sansom PI (1996). Synthesis of a new cylindrical calix[4]arene-calix[4]pyrrole pseudo dimer. *Tetrahedron Letters*.

[70] Kumar M, Bhalla V (2001). Synthesis of *p-tert*-butylcalix[4]arenes with diester bridge spanning the 1,3-(distal) positions on the lower rim. *Supramolecular Chemistry*.

[71] Arnecke R, Böhmer V, Ferguson G, Pappalardo S (1996). Inherently chiral derivatives of calix[5]crowns. *Tetrahedron Letters*.

[72] Saiki T, Goto K, Tokitoh N, Goto M, Okazaki R (2000). Syntheses and structures of novel *m*-xylylene-bridged calix[6]arenes: stabilization of a sulfenic acid in the cavity of calix[6]arene. *Journal of Organometallic Chemistry*.

[73] Geraci C, Consoli GML, Piattelli M, Neri P (2004). Doubly bridged calix[8]crowns. *Collection of Czechoslovak Chemical Communications*.

[74] Geraci C, Chessari G, Piattelli M, Neri P (1997). Cation encapsulation within a ten-oxygen spheroidal cavity of conformationally preorganized 1,5-3,7-calix[8]bis-crown-3 derivatives. *Chemical Communications*.

[75] Matthews SE, Schmitt P, Felix V, Drew MGB, Beer PD (2002). Calix[4]tubes: a new class of potassium-selective ionophore. *Journal of the American Chemical Society*.

[76] He Y-B, Huang H, Meng L-Z, Wu C-T, Yu T-X (1999). New calix[4]crown diacylamides with fluorescent response to complexation with metal ions. *Chemistry Letters*.

[77] Cram DJ, Cram JM (1978). Design of complexes between synthetic hosts and organic guests. *Accounts of Chemical Research*.

[78] Brouwer EB, Udachin KA, Enright GD, Ripmeester JA (2000). Amine guest size and hydrogen-bonding influence the structures of *p-tert*-butylcalix[4]arene inclusions. *Chemical Communications*.

[79] Buschmann H-J, Mutihac L, Jansen K (2001). Complexation of some amine compounds by macrocyclic receptors. *Journal of Inclusion Phenomena and Macrocyclic Chemistry*.

[80] Linnemayr K, Schmid ER, Allmaier G (1997). Characterization of calixarenes by positive- and negative-ion Californium-252 plasma desorption mass spectrometry. *Rapid Communications in Mass Spectrometry*.

[81] Arduini A, Ghidini E, Pochini A (1988). *p-t*-Butylcalix[4]arene tetra-acetamide: a new strong receptor for alkali cations [1]. *Journal of Inclusion Phenomena and Macrocyclic Chemistry*.

[82] Chang S-K, Jang MJ, Han SY, Lee JH, Kang MH, No KT (1992). Molecular recognition of butylamines by calixarene-based ester ligands. *Chemistry Letters*.

[83] Beer PD, Chen Z, Gale PA (1994). Cation recognition by new diester- and diamide-calix[4]arenediquinones and a diamide-benzo-15-crown-5-calix[4]arene. *Journal of Inclusion Phenomena and Macrocyclic Chemistry*.

[84] Beer PD, Chen Z, Gale PA (1994). Diester-calix[4]arenediquinone complexation and electrochemical recognition of group 1 and 2, ammonium and alkyl ammonium guest cations. *Tetrahedron*.

[85] Ungaro R, Pochini A, Andreetti GD, Domiano P (1985). Molecular inclusion in functionalized macrocycles—part 10^*^: crystal and molecular structure of a *p-tert*-butylcalix [6] arene
hexapodand. *Journal of Inclusion Phenomena and Macrocyclic Chemistry*.

[86] Ungaro R, Pochini A, Andreetti GD, Ugozzoli F (1985). Molecular inclusion in functionalized 
macrocycles—part 12: crystal and molecular structure
of a*p*-(1,1,3,3)-tetramethylbutylcalix[8]arene octapodand. *Journal of Inclusion Phenomena and Macrocyclic Chemistry*.

[87] Harris S, Diamond J, Diamond D, Barrett G, McKervey MA (1997). *IR Pat. Appl.*.

[88] Grady R, Butler T, MaCraith B, Diamond D, McKervey MA (1997). Optical sensor for gaseous ammonia with tuneable sensitivity. *The Analyst*.

[89] Loughran M, Diamond D (2000). Monitoring of volatile bases in fish sample headspace using an acidochromic dye. *Food Chemistry*.

[90] Calestani A, Minari P, Pochini A (1992). *Israel Journal of Chemistry*.

[91] Arnaud-Neu F, Fanni S, Guerra L (1995). Cation complexation by chemically modified calixarenes—part 7: transport of alkali cations by *p*-*tert*-butylcalix[*n*]arene esters and amides. *Journal of the Chemical Society, Perkin Transactions 2*.

[92] Beer PD, Drew MGB, Ogden MI (1997). First- and second-sphere co-ordination of a lanthanum cation by a calix[4]arene tetraamide in the partial-cone conformation. *Journal of the Chemical Society, Dalton Transactions*.

[93] Beer PD, Drew MGB, Leeson PB, Ogden MI (1995). Versatile cation complexation by a calix[4]arene tetraamide (L). Synthesis and crystal structure of [ML][CIO_4_]_2_ · *n*MeCN (M = Fe^II^, Ni^II^, Cu^II^,
Zn^II^ or Pb^II^). *Journal of the Chemical Society, Dalton Transactions*.

[94] Beer PD, Drew MGB, Kan M, Leeson PB, Ogden MI, Williams G (1996). Lanthanide structures, coordination, and extraction investigations of a 1,3-bis(diethyl amide)-substituted calix[4]arene ligand. *Inorganic Chemistry*.

[95] Beer PD, Drew MGB, Grieve A (1996). Neutral lanthanide di- and mono-meric complexes and selective extraction properties of a new 1,3-acid-diethyl amide substituted calix[4]arene ligand. *Chemical Communications*.

[96] Beer PD, Drew MGB, Leeson PB, Ogden MI (1996). Metal complexes of a calix[4]arene diamide: syntheses, crystal structures and molecular mechanics calculations on [Fe(L^1^-2H)][FeCl_4_] and [Er(L^1^-2H)(picrate)] (L^1^ = 5,11,17,23-tetra-*tert*-butyl-25,27-bis(diethylcarbamoylmethoxy)calix[4]arene). *Inorganica Chimica Acta*.

[97] Mckervey MA, Schwing-Weill MJ, Arnaud-Neu F, Gokek G (1996). Cation binding by calixarenes. *Comprehensive Supramalecular Chemistry. vol. 1*.

[98] Arena G, Contino A, Magri A, Sciotto D, Lamb JD

[99] Fanni S, Arnaud-Neu F, McKervey MA, Schwing-Weill M-J, Ziat K (1996). Dramatic effects of *p*-dealkylation on the binding abilities of *p*-*tert*-butylcalix[6]arenes: new
Cs^+^ and Sr^2+^ selective receptors. *Tetrahedron Letters*.

[100] Dozol JF, Garcia-Carrera A, Lamare V, Rouquette H Extraction of trivalent actinides and long-lived fission products by different classes of functionalized calixarenes.

[101] Cadogan F, Kane P, McKervey MA, Diamond D (1999). Lead-selective electrodes based on calixarene phosphine oxide
derivatives. *Analytical Chemistry*.

[102] Montavon G, Duplâtre G, Asfari Z, Vicens J (1996). Solvent extraction of sodium and potassium ions by a tetra-carboxylated calix[4]arene. *New Journal of Chemistry*.

[103] Ludwig R, Inoue K, Yamato T (1993). Solvent extraction behaviour of calixarene-type cyclophanes towards trivalent La, Nd, Eu, Er, and Yb. *Solvent Extraction and Ion Exchange*.

[104] Ohto K, Yano M, Inoue K (1995). Solvent extraction of trivalent rare earth metal lons with carboxylate derivatives of calixarenes. *Analytical Sciences*.

[105] Montavon G, Duplâtre G, Asfari Z, Vicens J (1997). Solvent extraction of uranium(VI) and thorium(IV) with a tetra-carboxylated calix[4]arene and effect of alkali ions (Na^+^, K^+^). *Solvent Extraction and Ion Exchange*.

[106] Nagasaki T, Shinkai S, Matsuda T (1990). Synthesis and solvent extraction properties of a novel calixarene-based uranophile bearing hydroxamate groups. *Journal of the Chemical Society, Perkin Transactions 1*.

[107] Nagasaki T, Shinkai S (1991). Synthesis and solvent extraction studies of novel calixarene-based uranophiles bearing hydroxamic
groups. *Journal of the Chemical Society, Perkin Transactions 2*.

[108] Agrawal YK, Sanyal M (1995). Separation and determination of uranium(VI) with calixarene hydroxamic acids. *Journal of Radioanalytical and Nuclear Chemistry*.

[109] Yordanov AT, Falana OM, Koch HF, Roundhill DM (1997). (Methylthio)methyl and (*N,N*-dimethylcarbamoyl)methyl upper-rim-substituted calix[4]arenes as potential extractants for Ag(I), Hg(II), Ni(II), Pd(II), Pt(II), and Au(III). *Inorganic Chemistry*.

[110] Arnaud-Neu F, Barrett G, Corry D (1997). Cation complexation by chemically modified calixarenes—part 10: thioamide derivatives of *p-tert*-butylcalix[4]-, [5]- and [6]-arenes with selectivity for copper, silver, cadmium and lead. X-Ray molecular structures of calix[4]arene thioamide-lead(II) and calix[4]arene amide-copper(II) complexes. *Journal of the Chemical Society, Perkin Transactions 2*.

[111] Koh KN, Imada T, Nagasaki T, Shinkai S (1994). Molecular design of hard-soft ditopic metal-binding sites on a calix[4]arene platform. *Tetrahedron Letters*.

[112] Arnand-Neu F, Böhmer V, Dozol J-F (1996). Calixarenes with diphenylphosphoryl acetamide functions at the upper rim. A new class of highly efficient extractants for lanthanides and actinides. *Journal of the Chemical Society, Perkin Transactions 2*.

[113] Malone JF, Mars DJ, Mckervey MA (1995). Calix[*n*]arene phosphine oxides. A new series of cation receptors for extraction of europium, thorium, plutonium and americium in nuclear waste treatment. *Journal of the Chemical Society, Chemical Communications*.

[114] Yordanov AT, Mague JT, Roundhill DM (1995). Solvent extraction of divalent palladium and platinum from aqueous solutions of their chloro complexes using an
*N,N*-dimethyldithiocarbamoylethoxy substituted calix[4]arene. *Inorganica Chimica Acta*.

[115] Yordanov AT, Roundhill DM, Mague JT (1996). Extraction selectivites of lower rim substituted calix[4] arene hosts induced by variations in the upper rim substituents. *Inorganica Chimica Acta*.

[116] Shortreed M, Bakker E, Kopelman R (1996). Miniature sodium-selective ion-exchange optode with fluorescent pH chromoionophores and tunable dynamic range. *Analytical Chemistry*.

[117] Kim JS, Yu IY, Suh AH, Ra DY, Kim JW (1998). Novel calix[4]arene azacrown ether. *Synthetic Communications*.

[118] Kim JS

[119] Kim JS, Shon OJ, Ko JW, Cho MH, Yu IY, Vicens J (2000). Synthesis and metal ion complexation studies of proton-ionizable calix[4]azacrown ethers in the 1,3-alternate conformation. *Journal of Organic Chemistry*.

[120] Sachleben RA, Urvoas A, Bryan JC, Haverlock TJ, Moyer BA, Hay BP (1999). Dideoxygenated calix[4]arene crown-6 ethers enhanced selectivity for caesium over potassium and rubidium. *Chemical Communications*.

